# Safety and efficacy of edaravone for patients with acute stroke: A protocol for randomized clinical trial

**DOI:** 10.1097/MD.0000000000024811

**Published:** 2021-02-26

**Authors:** Hailei Shan, Guangmei Jiao, Xi Cheng, Zhijie Dou

**Affiliations:** Department of Neurology, Affiliated Hospital of Chengde Medical University, Hebei, China.

**Keywords:** acute stroke, edaravone, efficacy, protocol, safety

## Abstract

**Background:**

We performed a randomized clinical trial protocol to assess the effectiveness of edaravone for acute stroke. We hypothesized that edaravone is beneficial in improving neurological impairment resulting from acute stroke.

**Method:**

The protocol was reviewed and approved by the Research Ethics Board of Affiliated Hospital of Chengde Medical University (0092-2394), each participant signed a written consent before participating, and SPIRIT guidelines were followed throughout. The inclusion criteria for patients were as follows: diagnosed as acute stroke (ischemic stroke or intracerebral hemorrhage) by head CT or MRI within 72 hours; age greater than 18; motor function disorder; Glasgow Coma Scale greater than 12. Patients with the following symptoms were excluded: concurrent serious complications, such as coma, drug allergy, mental disorder, and other severe organic lesions in the brain. Sixty patients were finally included in the study. The control group accepted conventional treatment, while the treatment group received edaravone treatment on top of the conventional treatment of the control group. After treatment, the differences in functional movement, living ability score, neurological score, treatment effect, and adverse reaction of these 2 groups were tested and compared.

**Discussion:**

As aging worsens, the incidence of acute stroke continues to increase. Brain damage will induce the production of oxygen radicals, which can damage the cytomembrane of brain cells and finally damage the nervous system and cause cerebral injury as well as the cerebral edema. Edaravone is an antioxidant and oxygen radical scavenger that can inhibit lipid peroxidation during the scavenging of oxygen free radicals. Besides, it can also elicit anti-inflammatory protective effects for nerve cells, increase cerebral blood flow volume, prevent the aggravation of cerebral hypoperfusion toward necrosis, reduce nerve damage, and improve neurological functions and prognosis. This is the first randomized controlled trial to assess the efficacy of edaravone for treating acute stroke. High quality, large sample size, multicenter randomized trials are still required.

**Trial registration:**

researchregistry6492.

## 1 Introduction

Stroke is the second most common cause of death and leading cause of adult disability worldwide.^[1–3]^ As life expectancy increases, the stroke burden worldwide is likely to increase, especially in the low-income and middle-income countries. Stroke seriously endangers the health and lives of people, and imposes a heavy and huge mental, economic and material burdens on the patients, their families and society.^[4,5]^

Reactive oxygen species are important contributors to brain injury after stroke, and free radicals are pivotal contributors to brain injury.^[6]^ Edaravone (MCI-186,3-methyl-1-phenyl-2-pyrazolin-5-one), a novel free radical scavenger, inhibits activation of lipoxygenase pathway in the arachidonic acid cascade and peroxidation of the phosphatidylcholine liposomal membrane in vitro.^[7]^ Thus, edaravone may provide effective neuroprotection, prevent vascular endothelial injury, and delay neuronal death in transient cerebral ischemia and ischemic stroke. It has been marketed in Japan by Mitsubishi Pharma as the first free radical scavenger for clinical use in the management of acute ischemic stroke since 2001. The reported neuroprotective mechanisms of edaravone are as follows:

1. quenching hydroxyl radical (OH), inhibiting OH-dependent and OH-independent lipid peroxidation;
2. inhibiting both water-soluble and lipid-soluble peroxyl radical-induced peroxidation systems; and
3. inhibiting both non-enzymatic lipid peroxidation and lip-oxygenase pathways.^[8,9]^

Therefore, edaravone has a powerful antioxidant effect on ameliorating ischemia or reperfusion-induced vascular endothelial cell injury and delayed neuronal death, attenuating brain edema and concomitant neurological deficits.^[10]^ However, previous systematic reviews for acute stroke found no conclusive evidence of its efficacy. To establish a definite evidence, we performed a randomized clinical trial protocol to assess the effectiveness of edaravone for acute stroke. We hypothesized that edaravone is beneficial in improving neurological impairment resulting from acute stroke.

## 2 Methods

### 2.1 Design

The protocol was reviewed and approved by the Research Ethics Board of Affiliated Hospital of Chengde Medical University (0092-2394), each participant signed a written consent before participating, and SPIRIT guidelines were followed throughout. The study was registered in the public trial registry (research registry 6492).

The randomization sequence was generated by an independent statistician using computer. The statistician provided opaque envelopes containing randomization assignments to the project nurse who was blinded to study group until opening the envelope. After the envelope was opened and the patient assigned to the intervention or control group, neither the nurse nor the participant were blind to the study group allocation. However, the research assistant who collected outcome measures data was blinded to study group assignment.

### 2.2 Participants

The inclusion criteria for patients were as follows: diagnosed as acute stroke (ischemic stroke or intracerebral hemorrhage) by head CT or MRI within 72 hours; age greater than 18; motor function disorder; Glasgow Coma Scale greater than 12. Patients with the following symptoms were excluded: concurrent serious complications, such as coma, drug allergy, mental disorder, and other severe organic lesions in the brain.

### 2.3 Treatment

Conventional treatment method was used for the control group: 80 mg of ligustrazine injection (drug specification: chemical medicine; 2 ml: 40 mg; Beijing Yanjing Pharmaceuticals Co., Ltd., China) +250 ml of 0.9% sodium chloride solution was intravenously injected once per day. Afterward, 100 mg of aspirin enteric-coated tablets (drug specification: chemical medicine; 100 mg; Bayer Healthcare Co., Ltd., Hong Kong) was administered orally before sleep. Basic treatment procedures, including dehydration and intracranial pressure drop, blood pressure and glucose regulation, and water-electrolyte imbalance correction, were performed according to the disease severity of the patients. Edaravone (drug specification: chemical medicine; 20 ml: 30 mg; Xi’an Lijun Pharmaceuticals Co., Ltd., Xi’an, China) was given to the treatment group based on the treatment for the control group. In particular, 30 mg of edaravone added to 100 ml of 0.9% sodium chloride solution was intravenously injected twice per day, and this procedure was completed within half an hour. Both treatments were given for 2 weeks, and treatment methods, such as solution expansion, anticoagulation, thrombolysis, or use of other drugs, which might influence the therapeutic effect, were prohibited during the treatment.

### 2.4 Observational indexes

Adverse reactions: during drug treatment, possible adverse drug reactions were observed and recorded, and hepatic and renal function analyses and routine blood and urine examinations were carried out after the treatment period.^[11]^

Daily living ability and movement function: after 2 weeks, the Barthel scale of activities of daily living was used to evaluate the patients’ living qualities. A high score corresponded to enhanced living quality.^[12]^

Neurologic impairment degree: The National Institutes of Health Stroke Scale (NIHSS) was used to evaluate the neurological impairment of patients before the treatment, in the first week of the treatment, and in the second week of the treatment. A high NIHSS score implied a serious neurological impairment.^[13]^

### 2.5 Efficacy criteria

Efficacy of edaravone was evaluated according to the clinical symptoms and manifestations of the patients and their NIHSS scores. Recovery criteria: clinical symptoms disappeared, the NIHSS score reduced to within 91% to 100%, and invalidism degree with grade 0; excellent efficient: clinical symptoms evidently improved, the NIHSS score reduced to within 46% to 90%, and invalidism degree within grades 1 to 3; partially efficient treatment: clinical symptoms slightly relieved, the NIHSS score reduced to within 18% to 45%, and patients basically recovered their self-care ability of daily living; ineffective treatment: clinical symptoms were not improved at all and even aggravated, and the NIHSS score reduced to not greater than 17%. The total treatment efficiency was calculated as follows: edaravone efficacy = recovery percentage + excellence efficient + partially efficient.

### 2.6 Statistical Analysis

Through utilizing the Microsoft Excel 2013, the data is recorded, and the analysis of all the data is carried out through utilizing IBM SPSS Statistics for Windows, version 20 (IBM Corp., Armonk, NY). With the χ^2^-tests and independent t-tests, the analysis for continuous variables and categorical variables are implemented respectively. All data are described with proper features, such as percentage, average and median. *P* value less than .05 indicates that there is statistical significance.

## 3 Results

Table 1 reflected the comparison of daily living ability, functional movement scores, NIHSS scores and adverse effects of the 2 groups before and after treatment.

**Table 1:** Comparison of daily living ability, functional movement scores, NIHSS scores and adverse reaction of the 2 groups before and after treatment.

Outcomes	Edaravone group (n = 30)	Control group (n = 30)	*P* value
ADL score Before treatment After treatment			
Functional movement scores			
Before treatment			
After treatment			
NIHSS scores			
Before treatment			
After treatment			
Edaravone efficacy			
Adverse reaction			

ADL = activities of daily living, NIHSS = National Institutes of Health Stroke Scale.

## 4 Discussion

As aging worsens, the incidence of acute stroke continues to increase.^[14,15]^ Brain damage will induce the production of oxygen radicals, which can damage the cytomembrane of brain cells and finally damage the nervous system and cause cerebral injury as well as the cerebral edema.^[16]^ The traditional treatments can improve the clinical symptoms of patients in some extents. However, it lacks strong prognostic reliability.

Edaravone is an antioxidant and oxygen radical scavenger that can inhibit lipid peroxidation during the scavenging of oxygen free radicals.^[17]^ Edaravone has a low molecular weight, so it can penetrate the blood–brain barrier easily. There are lipophilic groups in the molecular structure of edaravone, which is beneficial for it to scavenge a large amount of oxygen free radicals formed in the ischemic penumbra in brain tissues during ischemic–reperfusion process and can stimulate prostaglandins.^[18]^ It can inhibit the activation of hypoxanthine oxidase and xanthine oxidase, thereby reducing the accumulation of inflammatory mediators, such as leukotrienes.^[19]^ Besides, it can also elicit antiinflammatory protective effects for nerve cells, increase cerebral blood flow volume, prevent the aggravation of cerebral hypoperfusion toward necrosis, reduce nerve damage, and improve neurological functions and prognosis.^[20]^ This is the first randomized controlled trial to assess the efficacy of edaravone for treating acute stroke. High quality, large sample size, multicenter randomized trials are still required.

## Author contributions

Zhijie Dou planned the study design. Guangmei Jiao reviewed the study protocol. Xi Cheng will recruit participants and collect data. Hailei Shan wrote the manuscript. All of the authors have read, and contributed to the submitted manuscript.

**Conceptualization:** Guangmei Jiao.

**Data curation:** Guangmei Jiao.

**Formal analysis:** Xi Cheng.

**Funding acquisition:** Zhijie Dou.

**Investigation:** Xi Cheng.

**Writing – original draft:** Hailei Shan.
